# Implicit Theory of Mind – An overview of current replications and non-replications

**DOI:** 10.1016/j.dib.2017.11.016

**Published:** 2017-11-08

**Authors:** Louisa Kulke, Hannes Rakoczy

**Affiliations:** a Göttingen University, Leibniz ScienceCampus Primate Cognition, Göttingen, Germany

**Keywords:** Implicit Theory of Mind, Replications, Replication crisis

## Abstract

The current dataset contains a qualitative summary of (non-)replication studies of implicit Theory of Mind paradigms. It summarizes for each paradigm, how many replications, partial replications and non-replications were identified and how many of them were published or unpublished. Furthermore, descriptive data and sample sizes are reported. The dataset provides a qualitative overview of the published and unpublished findings in implicit Theory of Mind research.

**Specifications Table**

**Table:** 

Subject area	*Psychology*
More specific subject area	*Implicit Theory of Mind*
Type of data	*Table/Spreadsheet*
How data was acquired	*The data was collected by sending out emails containing a survey to the mailing list of the Cognitive Development Society (CDS,* cogdevsoc@lists.cogdevsoc.org*), and to colleagues, asking about any replications and non-replications (published or unpublished) that they had conducted of implicit Theory of Mind paradigms. Additionally, we did a literature search to add further published studies.*
Data format	*The data contains a qualitative summary of (non-)replication studies of implicit Theory of Mind paradigms, categorized by methods and original study. It summarizes for each paradigm, how many replications, partial replications and non-replications were sent to us and how many of them were published or unpublished. For each study we report the authors, whether the study was published, the replication type (direct or conceptual), the subject group tested, the total sample size, the sample size included in the final analyses, whether the original findings were replicated (and details if applicable), whether any novel conditions are analyses were implemented in the replication study, what methodological differences were reported, which exclusion criteria were applied, what results were reported and under which reference the study can be found (if any).*
Experimental factors	*The current dataset contains a qualitative collection of studies that was judged in regards to the principles mentioned above.*
Experimental features	*Published and unpublished studies were collected through a survey sent out to the mailing list of the Cognitive Development Society and to colleagues working on Theory of Mind. Additional studies were added after a literature research.*
Data source location	*The studies were collected in Göttingen, Germany. However, researchers from all over the world were contacted through the CDS mailing list.*
Data accessibility	*The Data is published alongside the article.*

**Value of the data**

• The dataset makes it possible for the reader to get an overview of published and unpublished Theory of Mind studies.
• Tables summarizing the number of successful, partial and unsuccessful replications of eleven different paradigms are provided.
• The reader can see how many subjects were tested for each study.
• In the light of the current replication crisis in psychology, this survey provides a qualitative overview of the current state of the field of implicit Theory of Mind research.

## 1 Data

The data contains a qualitative summary of (non-)replication studies of implicit Theory of Mind paradigms. It summarizes for each paradigm, how many replications, partial replications and non-replications were sent to us and how many of them were published or unpublished. Furthermore, we provide a summary of the sample sizes of replications, partial replications and non-replications of each paradigm. Although we carefully included all replication studies sent to us and all additional studies identified by us from the literature, the dataset merely presents a qualitative overview at the time of the survey and cannot guarantee that all published and unpublished replication studies were included. As furthermore preliminary findings were sent to us, we cannot guarantee that the reported findings were final, but they only reflect a preliminary picture and the reliability of each study should be carefully judged by the reader. The current paper does not contain a quantitative meta-analysis of the data but rather aims at providing a qualitative overview of implicit Theory of Mind replications.

In general, the survey contains five anticipatory looking false belief paradigms (by Southgate, et al. [1]/Senju, et al. [2], Surian and Geraci [3], Schneider, et al. [4], Low and Watts [5], and Clements and Perner [6]), two violation of expectation paradigms (by Onishi and Baillargeon [7], and Träuble, et al. [8]), three interactive paradigms (by Southgate, et al. [9], Buttelmann, et al. [10], and Rubio-Fernández and Geurts [11]) and one reaction time paradigm (by Kovács, et al. [12]).

For each replication study we report the authors, whether the study was published, the replication type (direct, i.e. using identical or identically structured stimuli to the original study, or conceptual, i.e. using different stimuli that differ in some features from the original ones; differences can be found under “Details” in the survey), the subject group tested, the total sample size, the sample size included in the final analyses, whether the original findings were replicated (and details if applicable), whether any novel conditions or analyses were implemented in the replication study, what methodological differences were reported, which exclusion criteria were applied, what results were reported and under which reference the study can be found (if any).

## 2 Experimental design, materials and methods

The data were collected by sending out emails containing a survey (Supplement A) to the mailing list of the Cognitive Development Society (CDS, cogdevsoc@lists.cogdevsoc.org, approximately 3700 members at the date) on the 22nd of August 2016 and a reminder on the 9th of May 2017, and to 17 colleagues, asking about any replications and non-replications (published or unpublished) that they had conducted of implicit Theory of Mind paradigms. Furthermore, we did a literature search to add further published studies. Only replications of implicit Theory of Mind tasks were included, while explicit tasks were excluded from the survey. We further did not include studies that only replicated true belief control conditions of false belief studies, studies that merely test implicit understanding of knowledge vs. ignorance rather than true Theory of Mind and studies on implicit understanding of desires rather than false beliefs.

For each paradigm for which we received responses we thoroughly read the original paper to identify the original results and specify them at the top of each table summarizing a paradigm. We further specified for each paradigm, which findings were counted as a “replication”, “partial replication” or “non-replication”.
