# Supplementary material for: Implicit Theory of Mind – An overview of current replications and non-replications
Source: Data Brief. 2017 Nov 8;16:101–4. doi: 10.1016/j.dib.2017.11.016 (PMC5694957; doi:10.1016/j.dib.2017.11.016)
Supplement: Supplementary file 2 — Supplementary material [file mmc2.rtf]

Study that was replicated/the task was based on:
____________________________________________________________________________

Measure
☐ Violation of Expectation
☐ Anticipatory Looking
☐ Interactive
☐ Other: _________________________________

Kind of replication and stimuli/procedures 
☐ Direct replication with original stimuli & procedure
☐ Conceptual replication with the following adaptations of stimuli and procedures: 
·	____________________________________________________________________________
·	____________________________________________________________________________

Conditions
[1] Original conditions
·	____________________________________________________________________________
·	____________________________________________________________________________
·	____________________________________________________________________________
[2] New conditions
·	____________________________________________________________________________
·	____________________________________________________________________________


Subject group (age, typically developing? etc.) & sample sizes: 
Subject group	Overall sample size	Subjects included	Subjects excluded	
				
				
				
…[add rows if necessary]…

Exclusion criteria: 
·	____________________________________________________________________________
·	____________________________________________________________________________

Main results:
[1] Replication of original analyses: 
·	____________________________________________________________________________
·	____________________________________________________________________________

[2] Novel analyses: 
·	____________________________________________________________________________
·	____________________________________________________________________________

Was the study pre-registered?
☐ Yes: _____________________________________________________________________
☐ No


What is the publication status of the study?
☐ published/in press at: _____________________________________________________________
☐ presented as a poster at: ___________________________________________________________
☐ unpublished data


Other commentaries:
____________________________________________________________________________
